# Population pharmacokinetics of fludarabine in patients with aplastic anemia and Fanconi anemia undergoing allogeneic hematopoietic stem cell transplantation

**DOI:** 10.1038/bmt.2017.79

**Published:** 2017-05-08

**Authors:** E Mohanan, J C Panetta, K M Lakshmi, E S Edison, A Korula, N A Fouzia, A Abraham, A Viswabandya, V Mathews, B George, A Srivastava, P Balasubramanian

**Affiliations:** 1 Department of Hematology, Christian Medical College, Vellore, India; 2 Department of Pharmaceutical Sciences, St. Jude Children's Research Hospital, Memphis, TN, USA

## Abstract

Although hematopoietic stem cell transplantation (HSCT) with a conditioning regimen consisting of fludarabine (F-araA) and cyclophosphamide (Cy) is associated with improved outcome in young patients with aplastic anemia (AA) and Fanconi anemia (FA), several factors limit the success of the procedure. We evaluated the population pharmacokinetics (POPPK) of F-araA and its influence on HSCT outcome in patients (*n*=53) with AA and FA undergoing HSCT. Patients carrying a 5′-UTR polymorphism in NT5E gene (rs2295890 G>C) exhibited significantly lower plasma F-araA clearance compared to those with wild-type genotype (7.12 vs 5.03 L/h/m^2^ (29%) *P*<0.05). F-araA clearance was significantly higher in patients with AA compared to FA (2.46 ×, *P*<1e−6). Of all the outcome parameters evaluated (engraftment, rejection/graft failure, GvHD, TRM, OS), high F-araA AUC (>29.4 μm*h) was the only significant factor associated with the development of aGvHD by both univariate and multivariate analysis (*P*=0.02). The influence of plasma F-araA levels need to be evaluated in a larger cohort of patients to propose the need for therapeutic drug monitoring.

## Introduction

Allogeneic hematopoietic stem cell transplantation (HSCT) is one of the curative modalities of treatment in patients with bone marrow failure conditions including aplastic and Fanconi anemia (FA). Cyclophosphamide (Cy)/anti-thymocyte globulin (ATG) is considered the standard conditioning regimen for patients with severe aplastic anemia (SAA) undergoing HSCT from a HLA-matched sibling donor,^1^ with bone marrow source and cyclosporine (CSA)/methotrexate (MTX) as GvHD prophylaxis for a long-term overall survival. However, graft rejection and GvHD still remain the major barriers. The introduction of a fludarabine (F-araA) based reduced intensity conditioning regimen has extended the availability of HSCT to patients who are older, heavily transfused and having delayed treatment from the time of diagnosis with HLA-matched related/unrelated donors.^2, 3, 4, 5^ The addition of F-araA to the conditioning regimen has been shown to provide additional immunosuppression for engraftment without increasing toxicity in patients with FA undergoing HSCT at our center.^4^ Also, conditioning with F-araA and Cy is associated with improved long-term survival compared to a historical cohort receiving Cy/ALG regimen in patients with SAA undergoing HSCT.^5, 6, 7, 8, 9^ However, aGvHD and transplant-related mortality (TRM) remains to be addressed to enhance the survival rate post HSCT in our cohort. Several variables including patient age, donor status, time from diagnosis to treatment and choice of conditioning regimen^5, 9, 10, 11, 12, 13^ contributes to the success of HSCT in these patients.

F-araA given IV as F-araA monophosphate is readily converted to F-araA (by the enzyme ecto-5′-nucleotidase or NT5E) which is taken up into the cells by nucleoside transporters (hENTs and hCNTs). Inside the cell, F-araA is phosphorylated by several kinases including deoxycytidine kinase (dCK), adenylate kinase (AK) and nucleoside diphosphate kinase (NDK) to its active metabolite F-araA triphosphate (F-araATP). All these genes have functional genetic polymorphisms affecting their expression or function and are implicated in severity of disease phenotype, cytotoxicity, cancer cell metabolism and metastasis^14, 15, 16, 17, 18, 19, 20^

Limited data available on the association between plasma F-araA levels and HSCT outcome showed higher plasma F-araA systemic exposure to be a risk factor for non-relapse mortality (NRM)^21^ or TRM.^22^ Phase I clinical studies suggested monitoring and dose adjustment of F-araA in patients with renal dysfunction^23, 24^ which was also demonstrated during HSCT condition.^22^ There has been no study comparing the F-araA pharmacokinetics (PK) and its effect on HSCT outcome in non-malignant hematological diseases. A recent study evaluated the influence of polymorphisms in genes (pharmacogenetics, PG) encoding enzymes/transporters involved in F-araA metabolic pathway on F-araA PK,^25^ but its influence on outcome has not been addressed. In this study we hypothesize that in addition to the known variables influencing the HSCT outcome in AA/FA cohort, F-araA PK and/or PG influences the HSCT outcome. We evaluated the population PK (POPPK) of F-araA and its influence on transplant outcome in a uniform cohort of patients with AA and FA undergoing HSCT.

## Patients and methods

### Patients

Patients diagnosed with AA or FA undergoing allogeneic HSCT at the Department of Hematology, Christian Medical College, Vellore between January 2012 and December 2014 receiving an F-araA based conditioning regimen were prospectively included in the study after obtaining written informed consent. This study was approved by the Institutional review board. All patients with AA received F-araA (30 mg/m^2^/day for 6 days over 1 h infusion from day −7 to −2) and cyclophosphamide (50 mg or 60 mg/kg/day for 2 days over 1-h infusion on day −3 to −2) prior to HSCT. Cyclosporine (2.5 mg/kg/dose, BD) and methotrexate or post-transplant cyclophosphamide (50 mg/kg/day × 2 days) was given as GvHD prophylaxis. Patients with FA received the same dose of F-araA, cyclosporine and methotrexate as that of AA, while the cyclophosphamide was given as 10 mg/kg/day × 2 days on day −3 to −2 and post-transplant cyclophosphamide was given at 25 mg/kg/day × 2 days as GvHD prophylaxis (Table 1).

### Reagents and chemicals

F-araA (Cat no: F2773) and the internal standard (IS) 5-flurocytidine (5-FC; Cat no: 543020) were purchased from Sigma-Aldrich, Bengaluru, India. The other reagents and chemicals N, N-Dimethylformamide, acetonitrile, ammonium acetate and acetic acid used were of Mass spectrometry grade from Fluka Analytical (Sigma-Aldrich Co., St Louis, MO, USA). Standards for F-araA assay were prepared in drug-free blank plasma (obtained from the Christian Medical College hospital blood bank).

### Analysis of plasma F-araA using liquid chromatography-tandem mass spectrometry (LC-MS/MS)

F-araA levels in plasma samples were measured by LC-MS/MS method using a Shimadzu-Prominence UFLC consisting of binary gradient pumps (LC-20AD), autosampler (SIL-HTc), mobile phase degasser (DGU20A_3_) and a column oven (CTO-20A) coupled with API2000 triple quadrupole mass spectrometer (ABSciex, MDS Sciex Inc., Toronto, ON, Canada). The system was managed using Analyst 1.4.2 software (ABSciex, Foster city, CA, USA). The mass spectrometry conditions were optimized using a separate external injection of 1000 ng/mL concentration of both pure F-araA and 5-FC at the rate of 10 μL/min. The parameters were adjusted to yield a maximum multiple reactions monitoring (MRM) signals (Supplementary Table S1). The Q1/Q3 for F-araA was set at 286.0/154.0 and 262.1/130.0 for internal standard, 5-FC in the positive ESI mode respectively. Chromatographic separation of the analyte was done using Syncronis C8 (2.1 × 50 mm, 5 μm, Thermo Scientific, Inc.) protected with a C8 guard column (10 × 2.1 mm, 3 μm) from the same source (Thermo Scientific, Inc., Madison, WI, USA). The LC conditions were as follows: Solvent A: 10 mm ammonium acetate (pH 5.0) and Solvent B: 100% acetonitrile was used as mobile phase with gradient elution of solvent B at 95% (0–0.5 min); 60% (0.5–2.0 min); 30% (2.0–4.0 min); 95% (4.0–7.0 min) at a flow rate of 0.25 mL/min. Retention time for F-araA was 1.26 min and the IS 1.10 min. The concentration of F-araA was expressed as ng/mL. The LLOQ was recorded to be 1 ng/mL and the method was linear for a wide concentration range from 7–7000 ng/mL (1–1000 μm) with mean *R*^2^ = 0.99±0.001 (Linearity, Accuracy and inter-day precision are as shown in Supplementary Table S2).

### Sample collection and processing

Peripheral blood (5 mL) was collected in sodium heparin tubes before the start (0 h) and 1, 2, 3, 5, 7 and 24 h after the infusion of F-araA, centrifuged immediately at 3000 r.p.m. for 5 min at 4 °C, plasma was separated and stored at −80 °C until further analysis. The F-ara-A PK samples were collected on HSCT days −7, −4, −3, −2; the PK sampling began with the start of F-araA administration. Series of F-araA standards (25 μL of 1–1000 μm) and 5-FC (25 μL of 250 ng/mL) were added to pre-labeled tubes containing 250 μL of drug-free blank plasma and vortexed thoroughly for 30 s. Ice cold acetonitrile was added and centrifuged at 13 000 r.p.m. for 25 min at 4 °C. The supernatants were dried under nitrogen gas at 40 °C. The residue was dissolved in 100 μL of mobile phase (10 mm Ammonium acetate pH 5.0 and 100% acetonitrile 9:1 v/v) and 10 μL was injected into the column. Patients' samples were prepared similarly except for an addition of 25 μL of deionized water instead of standard F-araA. The drug-free plasma spiked with three different concentrations (lower, middle and higher concentration) of pure F-araA were prepared and stored as controls/calibrators at −80 °C. These serve as quality controls during every run.

### Screening for polymorphisms in F-AraA metabolism and transport:

Genetic variants in the NT5E gene^17^ (rs2295890, rs9450278, rs9450279, rs4599602 and rs4458647) hENT1^26^ (rs747199), hCNT3^16^ (rs7853758) and NT5C2^27, 28^ (rs4917996) (with an allele frequency of >0.1 based on 1000 genome database) were screened in the HSCT patients using PCR followed by sequencing/RFLP analysis. The primer sequences and the PCR conditions are listed in Supplementary Table S3. The sequences were aligned using SeqScape software V2.6 (Applied Biosystems, Carlsbad, CA, USA) and the genotypes were documented.

### Chimerism analysis

Whole blood chimerism was evaluated by PCR amplification of the short or variable number of tandem repeats (STR/VNTR) markers followed by capillary electrophoresis (Genetic Analyzer ABI 3130) as reported previously.^29^

### F-araA PK and Population PK modeling

Non-linear mixed effects modeling analysis was performed with Monolix (version 4.3.3, LIXOFT, Batiment D, Antony, France) using the Stochastic Approximation Expectation-Maximization (SAEM) method. A two-compartment PK model was used to describe the data. The PK parameters estimated included clearance and volume (CL (L/h/m^2^) and V (L/m^2^)) along with the inter-compartmental rate constants (k12 and k21 (1/h)). Also, the individual *post hoc* parameter values were used to estimate the area under the concentration curve (AUC). The inter-individual and inter-day variability of the parameters was assumed to be log-normally distributed. A proportional residual error model was used with assumed normal distribution of the residuals.

The relationships between the PK parameters and covariates were described using the following model: θ=θ_Base_ × exp(β × covariate). A covariate was considered significant in the univariate analysis, if the addition of the covariate to the model reduced the objective function value (OFV) at least 3.84 units (*P*<0.05, based on the *χ*^2^-test for the difference in the −2 log-likelihood between two hierarchical models that differ by 1 degree of freedom).

### Limited sampling model

A limited sampling model (LSM) for F-araA PK was developed with these data to reduce sample collection time points for future studies. Specifically, we generated data sets from our original population with subsets of 3 or 4 time points per individual chosen from the original times (1, 2, 3, 5, 7 and 24 h after the infusion). Using the maximum, *a posteriori* probability (MAP) estimation approach in ADAPT V^30^ we then estimated the individual *post hoc* PK for each of the 3 and 4 timepoint per individual LSMs and compared the results to the individual *post hoc* PK estimated using all 6 time points per individual. The LSMs were ranked by their bias and error where bias was defined as: 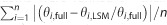 and error was defined as: 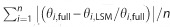 where *θ*_*i,*full_ are the individual PK parameter estimates using all 6 sample times and *θ*_*i,*LSM_ are the individual PK parameter estimates using the 3 or 4 timepoint LSMs.

### HSCT outcome

The influence of F-araA PK, PG and demographic factors on HSCT outcome parameters including engraftment, day 28 chimerism status, graft rejection, GvHD and overall survival, were evaluated. Neutrophil engraftment was defined as the ANC⩾500 × 10^6^/L on three consequent lab values; Complete chimerism was defined as ⩾95% of donor pattern in the patient’s peripheral blood and rejection was defined as more than 95% of recipient’s pattern. GvHD was defined according to Glucksberg criteria^31^ and the transplant-related mortality (TRM) as death (excluding death from relapse) occurring within 100 days from HSCT.

### Statistical analysis

Descriptive statistics were calculated for all variables. Statistical analyses were performed by Student's test, Mann–Whitney *U*-test, Kruskal–Wallis test and chi-squared analysis. Survival curves were drawn by the Kaplan–Meier method and compared by the log-rank test. The relationship of clinical features to the outcome of the procedure was analyzed by logistic regression and Cox regression analysis. The level of significance was set at 0.05 for all statistical tests. SPSS 16.0 software (SPSS Inc., Chicago, IL, USA) and GraphPad Prism (GraphPad software Inc., San Diego, CA, USA) were used for the analysis as appropriate. Overall the study was designed to have a power of 85% to see its effect on HSCT outcome, especially TRM and GvHD.

## Results

Forty patients with AA and 13 with FA were enrolled in the study. Patients received F-araA/Cy (*N*=29), F-araA/Cy/TBI (*N*=20) or F-araA/Cy/ATG (*n*=4) as their conditioning regimen. The patient demographics are given in Table 1.

### F-araA PK:

**Figure 1:**
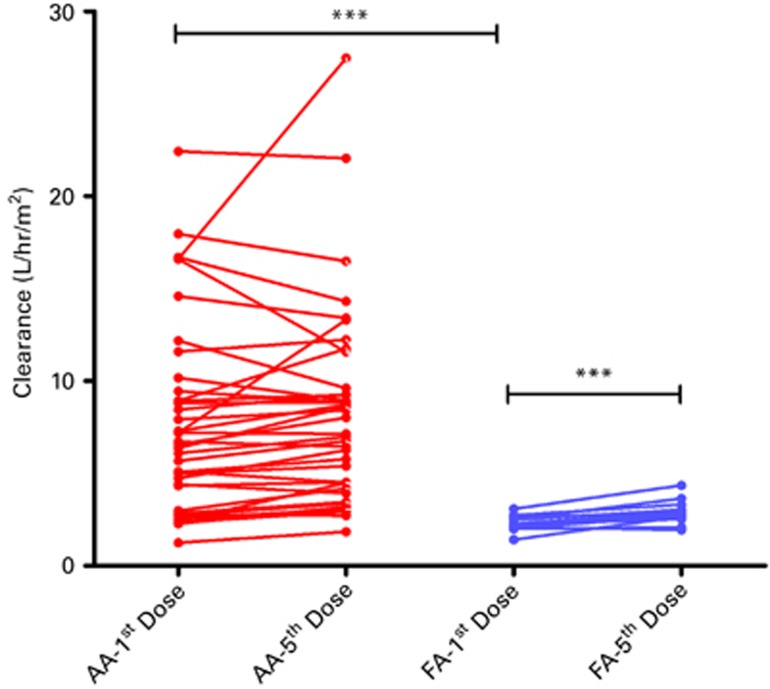
Inter-individual and inter-day variability in F-araA clearance in AA and FA patients. F-araA clearance was significantly higher in AA when compared to FA (*P*<0.0001). F-araA clearance is not different between the days in AA cohort, while the F-araA clearance on first dose is significantly lower than fifth dose in FA patients (*P*<0.0001); ***P<0.0001.

Samples for F-araA PK analysis were collected on day −7, −4 and −2 after the start of F-araA in 7 patients. For subsequent patients, samples were collected only on day −7 and day −3 after the start of conditioning to reduce the volume of blood being drawn during conditioning. The population PK model parameters for the base model which includes BSA normalized dose are summarized in Table 2. This model is significantly better than the model with non-normalized dose (the −2LL decreased 54.69 units; Table 3). The median *post hoc* estimated AUC for the first dose was 12.34 μm × h (3.63–52.47 μm × h) for AA and 29.76 μm × h (20.37–52.89 μm*h) for FA. There was considerable inter-individual variability (69 and 39% CV% for clearance and volume, respectively) in F-araA PK (Table 2). We also observed a significant inter- day variability (IDV) in the FA group but not in the AA group (Figure 1). Interestingly, the F-araA clearance was significantly higher in the AA group compared to patients with FA (median clearance 6.47 (range 1.24–22.43)L/h/m^2^ in AA vs 2.22 (range:1.41–3.08)L/h/m^2^ in FA; *P*< 0.0001; Figure 1).

### Influence of genetic variants on F-araA PK:

A single nucleotide polymorphism rs2295890 in the 5′-UTR region of the CD73/NT5E gene showed the expected/reported frequency of 0.18 in our study (Table 1). This SNP with a G>C change was found to be in complete linkage disequilibrium with four other SNPs in the same region namely, rs9450278, rs9450279, rs4599602, and rs4458647. Patients with variant genotype for rs2295890 (GC/CC) showed significantly lower plasma F-araA clearance compared to those with wild-type genotype (7.12 vs 5.03 L/h/m^2^; 29% *P*=0.038; Table 2).

Additionally, the parameters V and K_21_ increased significantly with respect to age (Table 2). The final population PK model including the covariates diagnosis (AA vs FA), rs2295890 (GG vs GC/CC), and age explained 46% of the inter-individual variability in clearance.

### Limited sampling model:

Based on the data from this group of individuals, we evaluated both 3- and 4-point LSMs (Supplementary Table S4). All the combinations of sampling times tested resulted in reasonable bias and error in the estimates of clearance with the 4-point sampling schedule of 1, 5, 7 and 24 h providing the smallest error.

### Influence of F-araA PK on HSCT outcome:

The outcome endpoints post HSCT are detailed in Table 4. Five of the 53 patients were not evaluable for analysis of HSCT outcome due to very early death (3 patients died prior to HSCT; two patients died before engraftment could be documented). Of the 48 evaluable patients, 45 (93.75%) engrafted at a median of 15 days post HSCT (range: 11–23 days) while 3 did not engraft (6.25%). Four had secondary graft failure while 8 of the 44 (18%) evaluable patients had mixed chimerism on day 28. Grade 2–4 acute GvHD was seen in 15 of the 46 evaluable patients (32.6%) with chronic GvHD in 16 of the 39 evaluable patients (41%). Grade 2–4 mucositis was observed in 33 (66%) patients, 4 (8%) developed grade 1 mucositis and the rest 13 did not have any toxicity. The development of GvHD or mucositis was not different between AA and FA cohort. The day 100 mortality was 30%, and 32 (60%) are alive at the last follow up. None of the PK parameters or demographic variables showed any association with engraftment, mixed chimerism, rejection, overall survival or TRM. Interestingly, patients with F-araA AUC more than the 75th percentile (6/10 with F-araA AUC >29.4 μm × h; vs 9/36 with F-araA AUC <29.4 μm × h had GvHD; *P*=0.057) showed trend to increased risk of developing acute GvHD. Multivariate analysis including the variables CD34+ cell dose, day of engraftment, GvHD Prophylaxis, ANC <15 days vs ⩾15, F-araA AUC >29.4 vs <29.4 μm × h, diagnosis (FA or AA), donor source as well the polymorphisms rs2295890, rs491799 on the incidence of acute GvHD showed F-araA AUC >29.4 μm × h to be significant factor (*P*=0.02).

## Discussion

HSCT with F-araA based conditioning regimen has been shown to have improved the overall and event-free survival (EFS) in patients with AA/FA who are young and those who failed immunosuppressive therapy.^2, 4, 32^ This is the first study demonstrating the PK of plasma F-araA in a uniform cohort of patients with AA and FA undergoing HSCT and also exploring the effect of polymorphisms in the genes encoding the enzyme NT5E involved in F-araA biotransformation. We established and validated a simple and rapid method to quantitate F-araA in human plasma. F-araA systemic exposure showed wide variation among the individuals (17-fold variation). Interestingly, patients harboring the variant genotype (GC, CC) of a 5′untranslated region SNP in the *NT5E* gene, rs2295890 exhibited a lower F-araA clearance when compared to patients with homozygous reference genotype (GG). Also, patients with AA exhibited significantly increased clearance of F-araA compared to those with FA, although both groups of patients have bone marrow failure.

PK of F-araA has been studied previously in various disease conditions^21, 22, 33, 34, 35, 36, 37^ and in various combinations. All the available F-araA PK studies have been in patients with a variety of malignant diseases and different doses including dose reduction of F-araA in patients with known renal dysfunction based on their creatinine clearance. This is the first study to address the F-araA exposure—response relationship in a uniform cohort of patients with AA and FA receiving a dose of 30 mg/m^2^/day F-araA. Table 5 summarizes the available literature on the PK of F-araA in patients undergoing HSCT. The dose of F-araA used and the PK parameters are comparable to the existing reports,^21, 22, 33, 34, 38^ we observed wide inter-individual variation in F-araA PK.

Interestingly, F-araA clearance was significantly higher in patients with AA compared to FA. There were patients in the AA cohort who also displayed F-araA clearance similar to those with FA. When we compared the demographic variables among the AA patients with F-araA clearance <3 L/h/m^2^ (equivalent to that of FA cohort) vs with those who had >3 L/h/m^2^, no significant difference was noted. We have observed comparable F-araA clearance in patients with Thalassemia^39^ and AML^40^ (unpublished observation) to that of the FA cohort. The increased clearance of F-araA in patients with AA needs to be explored further.

Another novel finding in this study is the impact of a 5′-UTR promoter polymorphism (rs2295890) in the NT5E gene encoding ectonucleotidase enzymes on the F-araA PK variability which has never been studied before. The subjects with variant genotype are shown to have a significantly lower clearance of F-araA than their counterparts with wild-type genotype. NT5E, also known as CD73 is a transmembrane glycoprotein, primarily involved in the purine salvage pathway. It is shown to have a multifaceted role in the normal physiology as well in the tumor context. The gene expression of NT5E has been studied as a prognostic marker in many solid tumors and hematologic malignancies. However, its role on drug metabolism is hardly known. A report by Li *et al.*^17^ has shown the effect of NT5E polymorphisms and its gene expression on the cytotoxicity of thiopurine drugs. The potential functional relevance of this polymorphism is currently being evaluated in our laboratory.

A 4 timepoint LSM was developed with this data to reduce sample collection time points for future studies. Specifically, we generated data sets from our original population and validated these results to the *post hoc* PK estimated using all available data. The LSM time points 1,5,7 and 24 h after infusion of F-araA were obtained with the least biased and error estimates. This model is comparable to the model developed by Salinger *et al.*^38^

Although appreciable inter-individual variability is observed in this cohort, none of the PK parameters are associated with HSCT endpoints such as engraftment, graft rejection, TRM and OS except aGvHD. Long-Boyle *et al.*^22^ in 87 patients with varied hematological disorders including AML, NHL or MDS showed that high F-araA exposure was associated with TRM and OS. McCune *et al*^31^ in 16 patients with malignant diseases and a high dose of F-araA (50 mg/m^2^) also observed an association between high plasma F-araA exposure and non-relapse mortality. It is possible that, since the incidence of events such as rejection or TRM is low in our cohort, none of the PK parameters is associated with these endpoints. Entirely different from the previous study by Long-Boyle *et al.*^22^ we observed a higher exposure of F-araA >29.4 μM × h to be significantly associated with the development of acute GvHD. Since the pathology of GvHD is still not exactly clear, one might think, enough ablation of the recipient lymphocytes and immunosuppression is necessary for preventing GvHD as well as rejection. Our data suggests that the F-araA PK has a therapeutic window beyond which organ damage, circumstantial infections, release of cytokine milieu occurs eventually leading to GvHD manifestation. The observation that there is an association of F-araA exposure and GvHD development needs to be explored further since the development of GvHD in the HSCT setting is multifactorial. Measurement of both plasma F-araA and intracellular F-araATP (which is the active metabolite exhibiting the cytotoxic profile of F-araA) would be ideal to understand the efficient lymphocyte depletion and a possible mechanistic explanation for the variation observed in the patients and associations with the outcome. However with the practical difficulty in the quantification of F-araATP as studied by McCune *et al.*,^35^ it would be interesting to study the kinetics of Cyclophosphamide (CY), which is administered along with F-araA in these patients which might bridge the gap in explaining the outcome endpoints. Further F-araA pharmacokinetics studies are warranted in a large uniform cohort of patients with various hematological disorders to arrive at the usefulness or need of therapeutic drug monitoring and personalizing the regimen.

## Figures and Tables

**Table 1:** Patient demographics

*Characteristics (N*=*53)*	*Median (Range)*
Age, yrs	17 (3–57)
Body weight, Kg	50 (12–89)
BSA, m^2^	1.49 (0.56–1.9)
Sex	35 males; 18 females
	
*Diagnosis*	
Aplastic anemia	40
Fanconi anemia	13
	
*Regimen*	
F-araA/Cy	29
F-araA /Cy/TBI	20
F-araA /Cy/ATG	4
	
*Donor source*	
Matched sibling donor	45
Alternate donor	8
	
*Stem cell source*	
Bone marrow	2
Peripheral blood	48
Not evaluable (died prior to T_x_)	3
CD34 cell dose (× 10^6^/kg)	9.8 (1.3–15.0)
	
*HLA Match*	
<8	6
⩾8	47
	
*GvHD* *prophylaxis*	
Cyclosporine/Methotrexate	32
Post T_x_ Cy	19
Not Evaluable	2
	
*SNP frequency in F-araA metabolic pathway genes*	
NT5E 5′-UTR rs2295890G>C	WT, GG-33
Not available=5	Var, GC/CC-15
CNT3 Exon 6 rs7853758G>A	WT, GG-32
Not available=7	Var, GA/AA-14
NT5C2 Intron rs4917996A>C	WT, AA-17
Not available=6	Var, AC/CC-30
hENT1 Exon 1 rs747199G>C	WT, GG-37
Not available=4	Var, GC/CC-12

Abbreviations: ATG=anti-thymoglobilin; Cy=cyclophosphamide; F-araA=fludarabine; SNP=single-nucleotide polymorphism; Var=variant genotype; WT=wild-type genotype; yrs=years.

Demographics of the patients undergoing HSCT with F-araA/Cy based conditioning regimen with or without TBI/ATG.

**Table 2:** Population pharmacokinetics of F-araA in Aplastic anemia and Fanconi anemia

*Parameter*	*BSA normalized*		*RSE (%)*		*Final model*		*RSE (%)*		P*-value*
CL (L/h/m^2^)	4.84		9.8		—		—		
WT, AA^a^	—		—		7.12		10		rs2295890: 3.8E−02; Diagnosis: 2.7E-07
WT, FA	—		—		2.90		17		
HET/MUT, AA	—		—		5.03		15		
HET/MUT, FA	—		—		2.05		19		
V (L/m^2^)	27.56		6.4		—		—		
									
Age on V	—		—		21.25^a^ exp (−0.013^a^ age)		11.8, 40.5		1.3E−02
k_12_ (h^−1^)	0.35		7.3		0.36		7.6		
k_21_ (h^−1^)	0.19		6.5		—		—		
Age on k_21_	—		—		0.14^a^ exp (0.016^a^ age)		10.1, 26.5		1.6E−04
σ prop (CV%)	0.19		3.6		0.19		3.6		
−2 Log-likelihood	312.34		2.4		262.51		2.1		3.9E−08
									
IIV, IDV	*(CV%)*		*RSE (%)*		*(CV%)*		*RSE (%)*		
CL	0.69	0.21	10.3	12.0	0.51	0.22	11.0	11.8	
V	0.39	0.21	13.1	16.4	0.36	0.22	13.8	15.8	
k_12_	0.37	0.18	17.5	36.5	0.41	0.19	15.4	35.7	
k_21_	0.26	0.23	28.7	30.1	0.14	0.20	65.6	34.7	

Abbreviations: CL=clearance; F-araA=fludarabine; HET=heterozygous variant; IDV=inter-day variability; IIV=inter-individual variation; MUT=mutant; RSE=residual error; WT=wild-type genotype.

a Covariate effect of rs2295890 and diagnosis on clearance. The covariates are defined as follows: rs2295890=(WT or HET/MUT); Diagnosis=(AA refers to Aplastic Anemia or FA-Fanconi Anemia).

**Table 3:** −2 Log-Likelihood of each covariate model tested

*Covariates included in model*	*−2 Log-Likelihood*
Non BSA normalized base model	376.03
BSA normalized base model	312.34
rs2295890	301.21
Age	299.16
Diagnosis	289.87
rs2295890, age	289.35
Diagnosis, age	281.38
Diagnosis, rs2295890	277.43
Final model: diagnosis, rs2295890, age	262.51

The categorical covariates are defined as follows: rs2295890=(0—WT or 1—heterozygous variant or Mutant); Diagnosis=(0—AA/SAA/VSAA (aplastic anemia) or 1—FA (Fanconi Anemia)). The covariates diagnosis and rs2295890 are included on the clearance and the covariate age is included on volume and the inter-compartmental parameter k_21_.

**Table 4:** HSCT Outcome

*Outcome parameters*	N	*(%)*	
*Engraftment*			
Yes	45	90	
No	5	10	
Not evaluable	3		
Day of engraftment (days)	15 (11–23)		
			
*Rejection*			
Yes	4	8.8889	
No	41	91.111	
Not evaluable	8		
			
*Chimerism*			
Complete	36	81.818	
Mixed	8	18.182	
Not evaluable	9		
			
*Mucositis*			
Yes	37	74	Grade 0–1: 17
No	13	26	Grade 2–4: 33
Not evaluable	3		
			
*aGvHD*			
Yes	15	32.609	Grade 0–1:31
No	31	67.391	Grade 2–4:15
Not evaluable	7		
			
*cGvHD*			
Yes	16	41.026	Grade 0–1:23
No	23	58.974	Grade 2–4:16
Not evaluable	14		
			
*TRM*^a^			
Yes	16	30.189	
No	37	69.811	

Abbreviations: aGvHD=acute GvHD; cGvHD=chronic GvHD; HSCT=hematopoietic stem cell transplantation; TRM=transplant-related mortality.

a The primary reasons for deaths were infection, GvHD and multi organ failure.

**Table 5:** Comparison of F-araA PK with previous reports

*Sl. no*	*Diagnosis*	*N*	*Conditioning regimen*	*F-araA dose*	*Type of donor*	*AUC (μm × h) median (range)*	*CL (L/h/m*^*2*^*) median (range)*	*Ref*
1	AML-05; CML-01; MDS-04; MF-05; CMML-01	16	F-araA: days −6 to −2; targeted daily IV Bu days −5 to −2;rATG on days −3 to −1	50 mg/m^2^/day	MRD-11 MUD-05	24.8 (16.3–39.9)	—	^21^
2	ALL-06; AML-26; NHL-17; MDS-14; HL-08; CML-01; Other-15	87	F-araA: days −6 to −2; Cy day −6	40 mg/m^2^/day (*N*=78) or 30-35 mg/m^2^/day (*N*=9)	MRD-22 MUD-65	40 mg/m^2^: 17.19 (7.02–40.35) 30-35 mg/m^2^ : 19.29 (15.08–24.56)	40 mg/m^2^: 16.0 (6.2–36.6)L/h; 30-35 mg/m^2^ : 11.5 (6.9–15.2) L/h	^22^
3	AML-05; MM-03; MDS-03; Others-05	16	F-araA: day −6 to −3; Bu: day −5 to −2	30 mg/m^2^/day	-	21.03 (10.17–38.56)	5.04 (2.7–10.2)	^33^
4	CML-04; MDS-38	42	F-araA : 4 days; Oral Bu : 4 days	30 mg/m^2^/day	MRD-16 MUD-26	Mean: 19.1 (s.d. 7.0) Range: 8.0–45.2	Mean: 6.3 (s.d. 2.4) Range : 2.2–13.3	^34^
5	NHL-34; CLL-22; AML-15; MDS-10; MM-09; Others-12	102	Flu: days −4 to day −2 2–4.5 Gy TBI	30 mg/m^2^/day	MRD-24 MUD-78	Mean±s.d. (Range) 19.6±4.80 (9.96–36.4)	—	^35^
6	NHL-05; HL-03; AML/MDS-03	11	Flu: days −6 to −2; CY days −6 and −5; TBI day −1	30 mg/m^2^/day	Haplo-11	16.4 (10.4–21.5)	—	^36^
7	MDS-18; AML-13; CML-05; CMML-02; MF-03	41	Protocol 1519 (*N*=27) Flu: days −9 to −6; targeted oral Bu days −5 to −2 Protocol 2041 (*N*=14) Flu: days −6 to −2, targeted daily IV Bu on days −5 to −2, and rATG IV on day −3, to −1	30 mg/m^2^/day 50 mg/m^2^/day	—	—	Protocol 1519: 9.1 (8–45.2); Protocol 2041: 7.07 (4.40–10.76)	^37^
8	AA-40; FA-13	53	Flu: day −6 to day −2 Cy: Day −3 and day −2	30 mg/m^2^/day	MSD-45 AD-08	AA-12.34 (3.63-52.47) FA-29.76 (20.37-52.89	AA-6.47(1.24–22.43) FA-2.22 (1.41–3.08)	Present study

Abbreviations: AA=Aplastic Anemia; AUC=area under the curve; FA=Fanconi Anemia; F-araA=fludarabine; PK=pharmacokinetics.

Dose and the PK parameters of AA was comparable to all the studies although the underlying disease and the combination of conditioning regimen are variable among the different studies.
