# Correction: Population pharmacokinetics of fludarabine in patients with aplastic anemia and Fanconi anemia undergoing allogeneic hematopoietic stem cell transplantation

**DOI:** 10.1038/s41409-018-0276-4

**Published:** 2018-08-22

**Authors:** E. Mohanan, J. C. Panetta, K. M. Lakshmi, E. S. Edison, A. Korula, N. A. Fouzia, A. Abraham, A. Viswabandya, V. Mathews, B. George, A. Srivastava, P. Balasubramanian

**Affiliations:** 1 Department of Hematology, Christian Medical College, Vellore, India; 2 Department of Pharmaceutical Sciences, St. Jude Children’s Research Hospital, Memphis, TN USA

**Keywords:** Diseases, Cancer

**Correction to:** *Bone Marrow Transplantation* 10.1038/bmt.2017.79; Article published online 8 May 2017.

This article was originally published under a CC BY-NC-ND 4.0 license, but has now been made available under a CC BY 4.0 license. The PDF and HTML versions of the article have been modified accordingly.

