# Supplementary material for: Population pharmacokinetics of fludarabine in patients with aplastic anemia and Fanconi anemia undergoing allogeneic hematopoietic stem cell transplantation
Source: Bone Marrow Transplant. 2017 May 8;52(7):977–83. doi: 10.1038/bmt.2017.79 (PMC5584518; doi:10.1038/bmt.2017.79)
Supplement: Supplementary Information [file bmt201779x1.docx]

**Supplementary Tables**

**Table S1: LC-MS/MS Conditions**

| **Mass spec conditions** | **values** |
| --- | --- |
| **Source/Gas parameters** |  |
| Curtain gas (CUR) | 10.0 |
| Collision Gas (CAD) | 5.0 |
| Ion Spray Voltage (IS)  Temperature (TEM)  Ion Source Gas 1 (GS1)  Ion Source Gas 2 (GS2) | 4500.0  400.0  50.0  40.0 |
| **Compound parameters** |  |
| Declustering potential (DP) | 40.0 |
| Focusing Potential (FP) | 400.0 |
| Entrance Potential (EP)  Collision Energy (CE)  Collision Cell Exit Potential (CXP) | 10.0  25.0  4.0 |

Mass spectrometry parameters and conditions for optimal intensity and MRM of analyte, F-araA and IS, 5-FC

**Table S2: F-araA analysis by LC-MS/MS- Method Validation parameters**

| Concentration (ng/mL) | Individual experiments | | | | | Mean | SD | Accuracy % | CV % |
| --- | --- | --- | --- | --- | --- | --- | --- | --- | --- |
|  | 1 | 2 | 3 | 4 | 5 |  |  |  |  |
| 10 | 10.32 | 9.97 | 9.29 | 9.69 | 8.60 | 10 | 1 | 95.75 | 6.94 |
| 50 | 52.16 | 52.18 | 45.44 | 45.87 | 45.16 | 48 | 4 | 96.33 | 7.61 |
| 100 | 111.28 | 107.07 | 107.15 | 104.36 | 89.25 | 104 | 9 | 103.82 | 8.20 |
| 200 | 227.06 | 214.85 | 200.24 | 199.43 | 206.15 | 210 | 12 | 104.77 | 5.52 |
| 500 | 492.37 | 497.96 | 485.83 | 507.73 | 491.00 | 495 | 8 | 99.00 | 1.68 |

The individual experiments on individual day (inter-day precision), accuracy and linearity is depicted. The inter-day CV% was <8.5%

**Table S3: Primer sequences and conditions**

| Gene/  Region | Forward sequence | Reverse Sequence | Product Size | Annealing temperature | SNPs Covered |
| --- | --- | --- | --- | --- | --- |
| NT5E/  5’UTR | ACATCCACAGCTTCAGTCCA | CTGTCCCTCTTTGAGCACCT | 458bp | 60^o^C | rs9450278  rs4599602  rs4458647 |
|  | TCTCAACCCAACAGGAAGCG | CGGACCCCTCCAATTCCTTC | 278bp | 54^o^C | rs2895890 |
| hENT1/  Exon 1 | CCCCTCCAATCTTCTTTTCAA | ACTTGACTGGGAGTTCACAGG | 1385bp | 58^o^C | rs747199 |
| hCNT3/  Exon 6 | CATCCTCCTCCATCTCCCTG | CTACTGAGGTTAGGGTGGGC | 228bp | 56^o^C | rs7853758 |
| NT5C2/  Intron | GTCTCGAACTCCCAACCTCA | GAGGGAGATGGTGGGTGTAC | 407bp | 58.5^o^C | rs4917996 |

The primer sequence for screening the 5 SNPs having more than 1% MAF in our population and its PCR conditions.

**Table S4**: **LSM Results.**

| Sample Times (hrs) | Bias (%) | Error (%) |
| --- | --- | --- |
| 3-sample LSM |  |  |
| 1, 3, 24 | 2.80 | 1.65 |
| 1, 2, 24 | 4.04 | 1.60 |
| 1, 7, 24 | 3.38 | 1.09 |
| 1, 5, 24 | 2.25 | -0.74 |
| 4-sample LSM |  |  |
| 1, 2, 7, 24 | 1.67 | 0.92 |
| 1, 3, 7, 24 | 1.92 | 0.86 |
| 1, 5, 7, 24 | 1.87 | -0.49 |

Clearance Bias and Error measures relative to the full sampling schedule (1, 2, 3, 5, 7, 24 hrs). In all cases the individual parameters were estimated using the MAP Bayesian estimation method.
